# 

**Published:** 1857-07

**Authors:** 


*Resignation.*—Prof. S. G. Armor has resigned his chair of Pathology and Clinical Medicine in the Medical College of Ohio. We have heard no name mentioned as likely to succeed him.

